# Medical and health science students' interpretation of miscommunication in simulated scenarios: the role of language, gender, and perceived professional authority

**DOI:** 10.3389/fmed.2026.1850742

**Published:** 2026-08-21

**Authors:** Mohammed Alzahrani, Yousef Hadhram

**Affiliations:** 1 English Language Department, College of Science and Health Professions, King Saud bin Abdulaziz University for Health Sciences, Riyadh, Saudi Arabia; 2 King Abdullah International Medical Research Center, Riyadh, Saudi Arabia; 3 Ministry of National Guard - Health Affairs, Riyadh, Saudi Arabia

**Keywords:** attribution theory, communication, identity, medical simulation, miscommunication

## Abstract

**Introduction:**

Little is known about how language, gender, and perceived professional authority simultaneously influence medical communication between doctors and patients. This study addresses this gap by examining Saudi medical and health science students' attribution of miscommunication in four medical simulation scenarios.

**Methods:**

Two groups of students observed two scenarios each, in which the agents' linguistic backgrounds, genders, and professional roles were systematically varied. Using a mixed-method design, students provided written responses to an elicitation task, followed by focus group and semi-structured interviews.

**Results:**

Blame for miscommunication was mostly attributed to patients. Lexical issues, communication strategies, and discourse were the main linguistic reasons cited, though further analysis of blame attribution by linguistic category and interlocutor provided more nuanced explanations.

**Discussion:**

Miscommunication is a complex process that goes beyond language, with gender and perceived professional authority playing a central role in how it is interpreted. Communication should be viewed as a shared, co-constructed responsibility, shaped by individuals' socially situated and perceived experiences.

## Introduction

According to Clark (1), language use is a joint action “that is carried out by an ensemble of people acting in coordination with each other” (p. 3). Through this communicative action, participants play dual roles as individuals and team members whose collective position is aimed at getting the message across as the sender intended. The process of communication, therefore, is a dynamic one. It develops from the active interaction of multiple elements that include information selection, expression, and interpretation (2). Since the interaction of these elements might result in either accurate comprehension or misinterpretation, they must be in sync with each other for the communication process to be successful. Similarly, miscommunication is also a complex process. Because language could be arbitrary, both the sender and receiver have to rely on contextual clues, prior knowledge, and cultural background to comprehend messages. However, while miscommunication could take place between members of the same group, it could also result from intercultural contact where “cross-cultural entanglements” signify interlocutors' social and cultural differences (3). Kecskes (4) noted that researchers have mistakenly attributed miscommunication to interculturality. He specified that the opposite might be true, noting that “the use of semantically transparent language by non-native speakers results in less misunderstandings and communication breakdowns than expected” (p. 139). This study does not aim to review communication theory broadly, but instead draws on interactional, social identity, and attribution-based frameworks most relevant to medical miscommunication, as detailed below.

### Theoretical and empirical background

Since medical communication is a complex process (5), doctors and patients might not consciously recognize miscommunication when it occurs (6). Mammas et al. (7) clarified that even among clinicians, medical miscommunication might result from the use of linguistically misleading terms. Similarly, Khalib and Farid (8) noted that communication barriers could be caused by the overreliance on medical jargon, poor listening abilities, and patients' reluctance to ask questions. Consequently, miscommunication can have downstream effects on care continuity (9). In addition, Lower English Proficiency (LEP) was reported to contribute to miscommunication, as LEP patients were considered more difficult to treat (10) and faced extra challenges because they tended, for example, to misunderstand discharge instructions (11). Non-verbal communication was reported to be similarly important especially if non-verbal cues disagreed with verbal statements (12).

Doctor-patient communication consists of three fundamental categories, which include content skills representing doctors' statements, process skills describing delivery methods, and perceptual skills that involve mental states and emotional responses during conversations (13). Others like Sahoo et al. (14) consider acceptance, comfort, responsiveness and empathy as essential elements for good communication between doctors and patients. Moreover, Mohd Salim et al. (15) noted that doctors and medical students reported effective eye contact, planning treatment, and ensuring privacy and confidentiality as essential constituents for effective communication. The patients, on the other hand, emphasized the importance of disease prognosis, doctor' empathy, and building and sustaining rapport. The basic principle of successful doctor-patient communication, therefore, entails expressing information in a way that allows both parties to understand the intended message, argued Gao et al. (16).

For such reasons, Klochko and Yashchenko (17) emphasized that future doctors need to be trained in professional communication by focusing on “teaching the situational and contextual adequate use of language” (p. 49). Non-verbal behaviors also need to be incorporated in the education and training of medical students (18). Another way to enhance medical communication could be through integrating it as a simulation-based module for doctors in training (5). Furthermore, as limited language skills may result in lower patient satisfaction and engagement, Ismaiel et al. (19) proposed peer-assisted learning programs pairing bilingual senior students with junior peers, to help the latter improve their communication with future patients in their first language. Although doctor-patient communication has been explored in prior research and was previously identified as requiring further investigation (20), it is still a complex topic today since both language and the profession are constantly evolving.

Beyond linguistic and interactional accounts, social identity processes also shape how miscommunication is perceived. Tajfel and Turner (21) argued that people's development of social connections originates from their membership within a group; a connection through which they are also inclined to compare their position to other groups. The development of identity also emerges through deductive and inductive processes, where existing group identities guide individual conduct, and individual actions help shape group identity (22). This becomes clear when examining communication in the medical field. Practicing physicians are trained to communicate in certain ways but also get a sense of their shared group identity when they encounter patients. Specifically, in the bottom-up or inductive process, medical miscommunication could be identified when “intragroup discussion creates a sense of group identity in the apparent absence of any direct intergroup comparison” [(22), p. 1]. Therefore, communication challenges become more evident when doctors start practicing their professions outside of their intergroup domain.

This process is also consistent with Bourdieu's concept of habitus, which he defined as “a system of lasting, internalized dispositions” [(23), p. 82]. Bourdieu's original analytical framework described how people internalize social structures by forming permanent internalized systems of “durable, transposable dispositions” (p. 72) that determine their perceptions and guide their behaviors. According to Bourdieu, people operate within social domains where economic, cultural, social, and symbolic capital each holds a unique value according to field-specific rules. Symbolic capital, specifically, stands for acquiring a reputation which demonstrates competence and displays respectability and honorability (24). Consequently, individuals' social positioning is influenced by such internalized rules (25, 26). Applying this to the medical field, miscommunication between doctors and patients might emerge as a result of mismatched habitus and unequal distribution of capital.

Prior research indicated that humans are highly sensitive to hierarchical structures because this capacity assists them in responding to social ranks (27, 28). One example lies in professional authority, defined by Harrits and Larsen (29) as “lay citizens” willingness to follow certain types of professional advice' (pp. 1015). Such authority is often connected to individuals' assessment of expertise and their perception of problems as “legitimate complexity” (i.e., problems perceived as too specialized for a layperson to resolve without expert input) that could be solved through professional advice (29). This applies to the medical field where doctors and nurses view their cooperation differently (30) and their role expectations not only differ but are also not openly discussed (31). Even among physicians, a hierarchical structure still exists in relation to one's chosen specialty (32), which clearly reflect different social status (33).

In his foundational work on naive psychology, Heider (34) introduced the attribution theory in which he considered individuals “naive psychologists” in the sense that they tend to justify actions made by others. In doing so, they attribute them either to internal dispositions like personality or external situations such as task difficulty. Weiner's later developments of the theory (35, 36) included the examination of people's perceptions about the causes of actions through causality, intentionality, and controllability. In addition, society depends on blame as an influential instrument which goes beyond psychological frameworks. It uses blame as a mechanism to establish right from wrong whenever negative events happen or rules get broken. Nadler and McDonnell (37) nonetheless noted that individuals tend to blame others for their actions “when they are confronted with a person who they think has a bad character even when the character information is totally unrelated to the action under scrutiny” (p. 255). In this study, attribution theory informs our analysis of how students assign blame for miscommunication to the doctor, the patient, or both, treating these attributions as evidence of dispositional vs. situational reasoning.

The practice of blame, therefore, functions to enforce accountability and maintain compliance. In doing so, power and social hierarchy play central roles in shaping how blame is being attributed (38, 39). Aquino et al. (40) reported that for personal offenses in the workplace, blame predicted greater revenge and less reconciliation. They noted that victims were more likely to seek revenge when offenders held lower status, and that lower-status employees were more inclined to retaliate. The process of assigning blame, accordingly, depends heavily on an individual's position within a social group as well as their biases. This is why discrimination against out-group members is central in the process of forming one' social identity (21). Consequently, the social classification of race, gender, socioeconomic status, and age might contribute to the creation of common stereotypes, representing collective assumptions about group members (41, 42).

Gender also stands as a crucial social identifier that determines how people assign blame (43). Prior studies clearly indicated that gender prejudice affected responsibility assessment in sexual assault cases (44–46). Strickler et al. (47) found that older and more politically conservative participants tended to blame the victim more than the perpetrator. More importantly, they revealed that women who exhibited sexist attitudes and scored higher on social dominance orientation (SDO) were more likely to assign blame on the victims. Gender also plays a significant role in healthcare communication (48, 49). However, there is a lack of research that explores the relationship between language, gender, and perceived professional authority, especially with regards to blame attribution in doctor-patient communications. Therefore, this study aims to fill this knowledge gap by examining medical students' attribution of miscommunication in different simulated medical scenarios.

In light of these different perspectives, the current study aims at exploring the roles of language, gender, and professional authority in shaping Saudi medical students' interpretation of miscommunication in simulated medical scenarios. This study is intended primarily for medical educators and health communication researchers involved in designing simulation-based curricula. Although the study mainly focuses on students' linguistic categorization of miscommunication (following the ten linguistic categories proposed by Shibata, (50)], it fills an existing gap related to understanding miscommunication beyond a sole focus on language. The study, therefore, is guided by the following questions:

1. How do medical students attribute blame for miscommunication in simulated medical scenarios based on agents' linguistic backgrounds, genders, and perceived professional authority?
2. What linguistic categories are highlighted in students' responses to these scenarios?
3. How is assigned blame for miscommunication distributed according to the emerging linguistic categories and perceived professional role?
4. How do students position themselves in response to these scenarios?

## Methodology

### Context and participants

The participants in this study were first-year preparatory-year (foundation year) students in the College of Science and Health Professions in a Saudi university. At this stage, students had not yet been streamed into specific medical or health science majors, as streaming occurs only after completion of the preparatory year. Due to the segregated nature of most education programs in Saudi Arabia, the study only included male students. During data collection, students were all enrolled in their second semester in university and had already passed all courses in their first semester. Additionally, data was collected in regular English classes right before the end of their second semester and shortly before they finished their first year. These English classes are a mandatory component of the standard preparatory-year curriculum for all students, taught in English, with Arabic as students' shared first language. This ensured that they understood the medical simulation scenarios, which were carefully created based on their levels and later validated by their responses in a post focus-group interview (described below). Since such classes normally consisted of small number of enrolled students, this nevertheless was convenient for the purpose of a medical simulation, which might not have been feasible with larger groups of students. To balance representativeness and feasibility, two groups were randomly selected for inclusion: Group 1 (*n* = 16) and Group 2 (*n* = 16). All ethical considerations were followed in this study, ensuring confidentiality of data and anonymity of participants.

### Medical simulation procedure

In each group, students observed two medical scenarios, each occurred between a doctor and a patient. Students were notified that the scenarios included incidents of miscommunication, and they were asked to identify responsible agents and any language-related causes for miscommunication. The medical simulation scenarios involved a patient showing up with abdominal pain followed by standard check-up procedures. In group 1, the first scenario included a male doctor and a female patient, both speaking English as a second language (L2). The second scenario was reversed, with a female doctor and a male patient, both being L2 English speakers. For Group 2, students also observed two medical scenarios; the first with one male doctor who spoke English as the first language (L1) and an L2 English female patient. The second scenario was reversed, with the doctor being a female L2 English speaker while the patient was a male L1 English speaker. Accordingly, we examined two conditions for each group. We specifically avoided the use of terms such as *native-speaker and non-native speaker* due to its controversial status in prior research that highlighted their unequal distribution of power (51, 52). Unlike Group 1, in which only the agents' language status varied between conditions, Group 2 varied both the physician's first-language status and gender simultaneously. This was an intentional design choice, allowing an exploratory, intersectional examination of language, gender, and professional authority together, consistent with the study's guiding research questions; the resulting interpretive constraints are discussed further in the Limitations section.

### Data collection and analysis

Through an elicitation task, students were asked to write as many reasons as possible for communication failures, focusing on blame attribution and linguistic reasons. In doing so, they were given the choice to provide their responses in their preferred language, whether Arabic or English. Each language-related written response was then analyzed and categorized according to blame attribution (specifically, whether the response identified the doctor, the patient, or both as responsible for the miscommunication) and according to ten linguistic categories (50): backgrounds, communication strategies, discourse, grammar, lexical, phonological, proficiency, speech style, speed, and translation. For linguistic categorization, responses that referred to more than one linguistic category were counted as separate ones for the linguistic analysis. The classification of responses within categories was done by two language-expert raters, yielding a Cohen's Kapp alpha coefficient of α = 0.87, which indicates strong consistency between the two raters. Any conflict between the raters, however, were discussed and solved to ensure an accurate classification of linguistic responses.

Simulation scripts were written by the researchers and piloted with participating agents who performed the roles of doctor and patient (hereafter, role-play confederates) prior to data collection. Simulations were conducted by the participating agents. Students did not know these participating agents, and therefore were led to believe the role-play confederates were real doctors and patients. Data collection took place in university classrooms, with class sizes ranging from 18 to 25 students. It is important to report that all participating confederates were invited to rehearsals and were trained twice before the scenarios; training involved reading through the scripts together, mock run-throughs, and feedback on delivery. Following the scenario and task, a focus-group interview was conducted with each group, and students were asked to complete a follow-up survey about their learning experience. On a separate day, 10 students (5 from each group) were randomly invited for follow-up semi-structured interviews, conducted by the researchers. Because all students shared several common characteristics, including similar age range, gender, and educational background, the purpose for this random selection was to obtain representative perspectives from each group. It is also noteworthy to emphasize that the main purpose of this study was not to generalize results as they are context specific. The focus, instead, was to examine each condition separately, and then, compare it to the other condition within the same group of participants. For data analysis, we categorized assigned blame for miscommunication based on professional role and gender. We also classified students' responses based on the aforementioned linguistic categories (50).

For students' follow-up interviews, thematic analysis (53) was used to further explore how students positioned themselves in response to the medical scenarios. The thematic analysis was conducted by the two authors following Braun and Clarke's six-step approach. Both authors independently reviewed and coded the interview transcripts during the initial stages of analysis. An initial codebook was developed inductively, based on recurring concepts and patterns identified in the first set of transcripts. This inductive process applied specifically to the coding of the follow-up interviews; the linguistic categorization of the written elicitation-task responses, by contrast, followed the ten predefined categories proposed by Shibata (50), and was therefore deductive. The authors then compared and discussed the preliminary codes to refine their code selection and resolve discrepancies. Both authors were involved in the qualitative analysis process and acknowledge that their academic backgrounds and prior experiences may have influenced data interpretation. To enhance analytical rigor and minimize potential bias, coding decisions and theme development were discussed collaboratively throughout the analysis process until consensus was reached. Due to the nature of the data, the quantitative and qualitative findings are presented separately. However, integration during the interpretation stage is found in the discussion section, where qualitative themes were used to explain and contextualize the quantitative results. Quotes from Arabic-language responses were translated into English by both authors, who translated independently and then used back-translation to check accuracy. Data were managed manually, without the use of qualitative analysis software. Participant member-checking was not conducted, as students were not accessible for follow-up after the data collection period concluded. Interview transcripts were coded individually and organized by theme content, following Braun and Clarke's inductive approach, rather than by which party the student blamed for the miscommunication. This mixed-method approach to data collection provided a deeper understanding of students' views with regards to blame attribution in medical simulated scenarios.

## Results

### Group 1: Blame attribution and linguistic categorization across conditions (L2 doctor–L2 patient interactions)

In Condition 1 (L2 male doctor, L2 female patient), 61 of 65 written responses were retained for analysis after excluding 4 irrelevant responses (Table 1). Students attributed blame for miscommunication primarily to the doctor (55.74%), followed by the patient (29.51%), with 14.75% assigning blame to both. In Condition 2 (L2 female doctor, L2 male patient), 49 of 56 responses were retained after excluding 7; attribution patterns shifted, with the patient assigned marginally more blame (48.98%) than the doctor (44.90%), and only 6.12% assigning blame to both.

**Table 1:** Blame attribution overview across conditions.

Condition	Doctor (#)	Doctor (%)	Patient (#)	Patient (%)	Both (#)	Both (%)	Total
Group 1, Condition 1	34	55.74%	18	29.51%	9	14.75%	61
Group 1, Condition 2	22	44.90%	24	48.98%	3	6.12%	49
Group 2, Condition 1	26	44.83%	28	48.28%	4	6.90%	58
Group 2, Condition 2	22	44.00%	24	48.00%	4	8.00%	50

Percentages are calculated within each condition's total retained responses. The # symbol denotes “number of responses” (i.e., a count), as distinct from the adjacent % columns.

**Figure 1:**
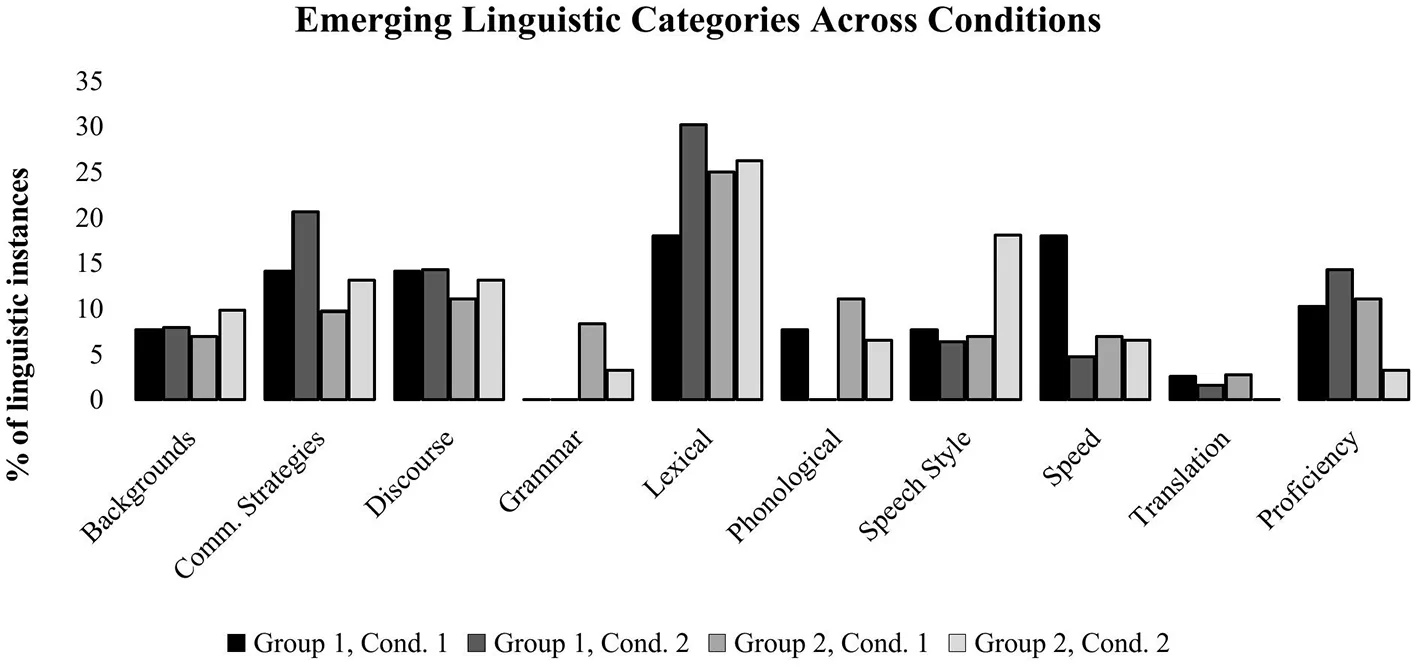
Emerging Linguistic Categories Across Conditions. Note: Percentages reflect the share of coded linguistic instances per condition (Group 1, Condition 1: n = 78 instances from 61 responses; Group 1, Condition 2: n = 63 instances from 49 responses; Group 2, Condition 1: n = 72 instances from 58 responses; Group 2, Condition 2: n = 61 instances from 50 responses).

Across both conditions, lexical issues were among the most frequently cited linguistic categories (tied with speed at 17.95% in Condition 1; the single most frequent category at 30.16% in Condition 2), while translation was consistently the least cited (2.56% and 1.59%, respectively); grammar did not appear at all in Condition 2 (Figure 1).

Blame attributed within categories (Table 2) was most concentrated for lexical and speed issues, both attributed almost exclusively to the doctor in both conditions (92.86%/100% in Condition 1; 73.68%/100% in Condition 2), while proficiency and communication strategies were attributed predominantly to the patient in both conditions (87.50%/72.73% in Condition 1; 88.89%/61.54% in Condition 2).

**Table 2:** Blame attribution to interlocutor within linguistic categories, by condition.

Linguistic Category	Group 1, Cond. 1			Group 1, Cond. 2			Group 2, Cond. 1			Group 2, Cond. 2		
	Dr	PT	Both	Dr	PT	Both	Dr	PT	Both	Dr	PT	Both
Backgrounds	50.00	16.67	33.33	0.00	40.00	60.00	60.00	20.00	20.00	16.67	66.67	16.67
Comm. Strategies	18.18	72.73	9.09	38.46	61.54	0.00	42.86	57.14	0.00	62.50	37.50	0.00
Discourse	27.27	45.45	27.27	44.44	55.56	0.00	37.50	62.50	0.00	37.50	50.00	12.50
Grammar	0.00	0.00	0.00	0.00	0.00	0.00	0.00	100.00	0.00	50.00	50.00	0.00
Lexical	92.86	0.00	7.14	73.68	26.32	0.00	72.22	27.78	0.00	62.50	31.25	6.25
Phonological	16.67	16.67	66.67	0.00	0.00	0.00	25.00	37.50	37.50	0.00	75.00	25.00
Proficiency	0.00	87.50	12.50	0.00	88.89	11.11	37.50	50.00	12.50	0.00	100.00	0.00
Speech Style	50.00	33.33	16.67	25.00	75.00	0.00	40.00	60.00	0.00	18.18	72.73	9.09
Speed	100.00	0.00	0.00	100.00	0.00	0.00	80.00	20.00	0.00	75.00	25.00	0.00
Translation	100.00	0.00	0.00	100.00	0.00	0.00	100.00	0.00	0.00	0.00	0.00	0.00

Dr, doctor; PT, patient; Both, shared attribution. Percentages are calculated within each linguistic category (i.e., of all responses coded under a given category, the percentage attributed to each interlocutor).

Examined separately by interlocutor (Table 3), the doctor was most often blamed for speed and lexical issues in Condition 1 (34.15%, 31.71%) and for lexical issues alone in Condition 2 (25.45%), while the patient was most often blamed for communication strategies and proficiency in both conditions (33.33%/29.17% in Condition 1; 25.81% each in Condition 2).

**Table 3:** Linguistic category distribution within each interlocutor's attributed blame, by condition.

Linguistic Category	Group 1, Cond. 1			Group 1, Cond. 2			Group 2, Cond. 1			Group 2, Cond. 2		
	Dr	PT	Both	Dr	PT	Both	Dr	PT	Both	Dr	PT	Both
Backgrounds	7.32	4.17	15.38	0.00	6.45	75.00	8.57	3.13	20.00	4.00	12.90	20.00
Comm. Strategies	4.88	33.33	7.69	9.09	25.81	0.00	8.57	12.50	0.00	20.00	9.68	0.00
Discourse	7.32	20.83	23.08	7.27	16.13	0.00	8.57	15.63	0.00	12.00	12.90	20.00
Grammar	0.00	0.00	0.00	0.00	0.00	0.00	0.00	18.75	0.00	4.00	3.23	0.00
Lexical	31.71	0.00	7.69	25.45	16.13	0.00	37.14	15.63	0.00	40.00	16.13	20.00
Phonological	2.44	4.17	30.77	0.00	0.00	0.00	5.71	9.38	60.00	0.00	9.68	20.00
Proficiency	0.00	29.17	7.69	0.00	25.81	25.00	8.57	12.50	20.00	0.00	6.45	0.00
Speech Style	7.32	8.33	7.69	1.82	9.68	0.00	5.71	9.38	0.00	8.00	25.81	20.00
Speed	34.15	0.00	0.00	5.45	0.00	0.00	11.43	3.13	0.00	12.00	3.23	0.00
Translation	4.88	0.00	0.00	1.82	0.00	0.00	5.71	0.00	0.00	0.00	0.00	0.00

Dr, doctor; PT, patient; Both, shared attribution. Percentages are calculated within each interlocutor's total attributed blame (i.e., of all instances blamed on the doctor, the percentage falling under each linguistic category).

### Group 2: Blame attribution and linguistic categorization across conditions (L1/L2 doctor–patient interactions)

In Condition 1 (L1 male doctor, L2 female patient), 58 of 61 written responses were retained for analysis after excluding 3 irrelevant responses (Table 1). Blame attribution was closely split, with the patient assigned slightly more blame (48.28%) than the doctor (44.83%), and 6.90% assigning blame to both. In Condition 2 (L2 female doctor, L1 male patient), 50 of 51 responses were retained after excluding 1; attribution remained similarly close, with the patient again assigned marginally more blame (48%) than the doctor (44%), and 8% assigning blame to both.

Across both conditions, lexical issues were consistently the most frequently cited linguistic category (25% in Condition 1; 26.23% in Condition 2), while translation was the least cited in Condition 1 (2.78%) and entirely absent from Condition 2 (Figure 1).

Blame attributed within categories (Table 2) was most concentrated for translation and speed, both attributed entirely to the doctor in Condition 1 (100% each), and for proficiency, attributed entirely to the patient in Condition 2 (100%).

Examined separately by interlocutor (Table 3), the doctor was most often blamed for lexical issues in both conditions (37.14%; 40%), while the patient's blame was most concentrated in grammar in Condition 1 (18.75%) and in speech style in Condition 2 (25.81%).

### Students' positioning in response to the medical simulation scenarios

The students' interviews highlighted additional aspects related to blame attribution and communication failures. For both groups, students had diverging views with regards to the influence of gender on medical communication. One student who believed there was no influence of gender remarked:

I don't think gender is something that came in the way. I think it's the person, their self, who defines the interaction. (Participant 1)

Another student emphasized professional behavior as an important skill for doctors, regardless of their gender:

In the end, it is about abdominal pain. So, whether the doctor is a man or a woman doesn't make a difference. It's about the behavior. It is true that there was some difficulty in communication, but body language played a good role in addition to the elaborated explanations. I think the situation was clear to me as an observer. (Participant 2)

Other students who thought gender had an influence on communication attributed it to gender-related emotions. For instance, a student commented:

One thing I noticed in the two conversations is the issue of gender. There has been always some kind of tension between males and females where a man might feel shy and can't express himself well. And the same goes for a woman. Men also avoid saying too much. But when they share the same gender, you can see more harmony and ease. (Participant 3)

While gender was important, lexical choices, speed, and communication strategies were essential language elements needed for effective communication:

To be honest, the core idea of misunderstanding was actually coming from the doctor. I mean he used difficult words and spoke quickly. So, he was not giving the patient a chance to respond and clarify her problem. And as for the patient, she wasn't clarifying her problem more clearly because the doctor was a man. (Participant 6)

On a similar note, another student suggested that doctors should use clear language considering

if the doctor is a native speaker and the patient is not, it's better to use clear and simple, everyday language. (Participant 4)

Indeed, it was clear that language was a barrier between doctors and patients who did not share the same first language:

That was obvious. It was definitely a cause [of miscommunication]. You're talking about two people who don't share a common language or a way to communicate. So how will the information reach the other party, especially when the other party, which is the doctor, needs to know important details in order to understand how to treat you and ease these painful symptoms? (Participant 2)

Furthermore, most students emphasized the higher professional roles of doctors. One student stated that both doctors and patients might stimulate miscommunication, but still acknowledged the higher position of doctors:

A doctor might make a mistake, and every human might make a mistake. But the doctor has higher experience, reputation, and knowledge, so the margin for error is lower. The patient might forget the medication schedule or the doctor's instructions, so they all make mistakes. (Participant 3)

Here, “they” is used generally to refer to both doctors and patients, rather than one specific party.

Another student confirmed this, stating that people tend to highlight doctors' authority as one that is almost flawless:

I mean, most people tend to place the blame on the patient more than the doctor, given that doctors have a certain allure and prestige due to their job and specialization, and that they are knowledgeable in the field, so no one expects a mistake from them. (Participant 4)

However, a student who believed that both the doctor and patient were equally held responsible for miscommunication indicated that communication should be a mutual action:

because I think there is no, like, someone who's 100% mistaken. I think they both should work together. (Participant 7)

Overall, students viewed the interaction between gender, language, and professional authority as a complex experience. Throughout the interviews, students held different beliefs and positions about miscommunication, yet they emphasized the important role of language for both doctors and patients. They also highlighted individual and biased opinions when it came to gender and noted the higher authority assigned to doctors.

## Discussion

Overall, the linguistic coding analysis revealed that the most frequent linguistic categories associated with miscommunication were lexical issues and speed, with communication strategies and discourse also featuring prominently in some conditions, while grammar and translation were largely overlooked by students. In other words, students perceived miscommunication as a problem of function and comprehension rather than structure. This is because categories such as lexical choice, communication strategies, and speed relate to how effectively meaning is conveyed and processed in real-time. In contrast, categories such as grammar and translation relate more to the formal structure of language itself. This goes in line with prior literature, which reported that the use of medical jargon, complicated language, and the failure to express ideas clearly might often lead to miscommunication (7, 54–56). The results are also consistent with Kecskes's (4) emphasis on the significance of both prior and actual situational experiences for comprehension and in the meaning-making process. Similarly in this study, it could be noticed that the lack of prior knowledge for the patient agent might have been perceived by students as related to lexical ambiguity, whereas their views on actual situational experiences might have been shaped by the absence of proper communication strategies and coherent discourse. For instance, when the patient agent lacked prior knowledge of medical terminology, students tended to frame this as a lexical issue (e.g., unfamiliar vocabulary), whereas breakdowns in the flow of the consultation itself were more often framed as issues of communication strategy or discourse coherence.

When analyzing attribution of miscommunication for doctors and patients under each category, it was noted that across conditions, doctors were consistently more blamed for lexical and speed issues while patients were assigned more blame for proficiency. This goes in line, to some extent, with Shibata's (50) analysis of the interaction between L1 and L2 English speakers in which she found that miscommunication due to speed was attributed to the L1 English speaker while proficiency was connected to the L2 English speaker. The results of this study, however, showed that in these specific categories, it was rather professional authority, not necessarily language, that influenced students' attribution of blame. This is supported by the consistency of these attribution patterns across both the L1 and L2 doctor conditions in Group 2, where blame for lexical and speed issues remained concentrated on the doctor regardless of whether the doctor's first language matched the patient's, which suggests that the doctor's professional role, rather than language background, was the more consistent driver of blame attribution. Additionally, by analyzing miscommunication separately for each interlocutor, the analysis indicated that doctors were once again assigned blame mostly for lexical issues and speed. The patients, nonetheless, were blamed primarily for discourse and lexical issues. As noted earlier, this again mirrors this pattern, in which lexical and speed issues were linked to the L1 English speaker, while lexical issues also prompted miscommunication for the L2 speaker.

As for professional authority, miscommunication was assigned mostly to patients, highlighting doctors' epistemic authority over patients' involvement (57, 58). The results, however, showed that students did not place the blame by large on patients even when an L1 speaker was added. This suggests that when English was the agents' first language, it did not essentially influence students' judgments, but rather their professional roles. Nevertheless, this pattern might depend on language and gender. To clarify, blame was mostly connected to male agents, except in the presence of an L1 English male doctor. Otherwise, students were more empathetic with female agents. The opposite occurred in the scenario that included a male L2 English doctor and L2 English patient. In this case, students blamed the doctor the most compared to other conditions. Furthermore, noting that male patients were assigned more blame, this implies that female doctors might have been viewed as more empathetic and attentive. This is possibly why phonological issues for female doctors were never reported by students. These findings echo previous studies that noted such characteristics to female physicians (58–60). It is important to note, however, that this finding should be interpreted with caution: because Group 2 varied both gender and first-language status simultaneously (see Limitations), the absence of phonological issues for female doctors cannot be attributed to gender alone, independent of the confounding shift in language background. These patterns also align with social identity theory (21), whereby students' group-based expectations of doctors and patients—shaped by professional and gender identities—appeared to guide their attribution of blame.

In her study of recorded interactions between a female gynecologist and pregnant patients, Rossi (48) noted that the dialogues were mainly cantered on patients, which led to “a co-construction of narratives in which personal uniqueness is more relevant than gender roles, expectations, and stereotyped representations” (p. 1125). However, when the husbands of such women took part in the conversations, the focus of communication shifted to the doctor. Rossi further noted, “and this usually leads to asymmetrical and directive constructions of narratives about differentiated and traditional gender roles” (p. 1125). In the current study, a similar theme emerged with the presence of a male L1 English doctor. By speaking English as a first language, being male, and practicing as a doctor, it is possible that the attribution of miscommunication by the students to the female patient (Group 2, Condition 1) was a result of not only gender, but the multifaceted roles that the doctor held. This reflects an intersectional pattern (61, 62), whereby overlapping identities—language background, gender, and professional role—combined to shape how blame was attributed, rather than any single identity acting in isolation.

By applying the attribution theory to the results (34, 36), the students appeared to prejudge doctors' languages, genders, and professional roles (dispositional) in communication while still considering, for example, their use of medical jargon (situational). Because doctors in this study held socially preapproved positions of authority, power, and accountability, they were thus expected to act in certain ways, confirming Bourdieu's views on symbolic capital (1986). Students also appeared to connect such authority to the idea of legitimate complexity (63), which refers in this context to the relationship between professional expertise (of doctors) and assessment of complex issues (by patients) (29). Students' views on medical miscommunication as legitimately complex and requiring an expert opinion might have influenced their evaluation and attribution of miscommunication. Therefore, the results propose a partial shift from patient-blaming discourse caused by language alone to considering how communication could be viewed beyond content (13) and more as a shared and socially situated responsibility (16).

The study, however, is limited by being context-specific, as its results may not be generalizable to other professions. The sample was also restricted to male students, due to the segregated nature of most education programs in Saudi Arabia, which limits the generalizability of the gender-related findings to mixed-gender or female-only populations. Additionally, in Group 2, both the doctor's gender and first-language status varied simultaneously between conditions; while this was an intentional design choice allowing an exploratory, intersectional look at these factors together, it also means that language and gender effects cannot be cleanly separated in this group, and findings involving Group 2 should be interpreted with this in mind. Besides, though students' views provided valuable insights into understanding medical miscommunication, they are subject to change over time, place, and experience. It is therefore pivotal to track any changes related to miscommunication through longitudinal research. Future work could also draw on comparative international evidence—for example, studies examining patient-provider language concordance and discordance in Australia (64) and Japan (65)—to situate these findings within a broader cross-national context. In addition, although the number of students in each group was relatively low, the small-scale medical simulations offered an immediate, real-time observational experience, which might not have been feasible in other conditions involving larger groups. Nonetheless, the study offers important implications. Both doctors and patients need to go beyond a sole focus on language structures and consider meaning and context as two inseparable constructs needed for successful communication. Also, communication barriers could be incorporated by educators into the design of medical curricula and simulation exercises. Yet, they need to view communication as a complex process that needs to be carefully considered. Likewise, medical students have to raise their awareness of the various factors that might trigger both patients and doctors' miscommunication. This includes the use of medical jargon and communication strategies that match potential patients' levels. Methodologically, future research could extend previous work on the attribution theory to explore communication in other professional fields. It could also examine different conditions involving, for example, same-gender or intergenerational communication and elaborate more on other multilingual contexts.

## Conclusion

This study examined students' linguistic attribution of miscommunication in different medical simulation scenarios. The findings revealed the complex dynamics of language, gender, and perceived professional authority in shaping students' interpretation of doctor-patient miscommunication. While agents' linguistic backgrounds (English or Arabic) played an important part, gender had a nuanced influence on how communication was assessed. In addition, professional authority played a major role that needs to be considered in future communication research. Overall, the findings underscore the importance of examining communication as a shared responsibility that is not only influenced by language but also by how speakers are socially situated and perceived. Although specialized training on medical communication might be helpful, health practitioners and medical educators need to consider the complexities of (mis)communication and avoid viewing it as a fixed phenomenon.

## Data Availability

The raw data supporting the conclusions of this article will be made available by the authors, without undue reservation.
